# Etoricoxib and its hidden risks: a case-based review of dermatological, hematological, and cardiovascular complications

**DOI:** 10.17179/excli2025-8751

**Published:** 2025-09-21

**Authors:** Mohamamd Anas Ansari, Arun Kumar

**Affiliations:** 1 School of Pharmacy, Graphic Era Hill University, Dehradun, Uttarakhand, India

**Keywords:** Stevens-Johnson syndrome, toxic epidermal necrolysis, fixed drug eruptions, atrial fibrillation, hypertension, etoricoxib

## Abstract

This review analyzes case reports of adverse drug reactions (ADRs) attributed to etoricoxib, with particular emphasis on Stevens-Johnson syndrome (SJS), toxic epidermal necrolysis (TEN), fixed drug eruptions (FDE), atrial fibrillation, hypertension, thrombocytopenia (TP), immune hemolytic anemia (IHA), acute generalized exanthematous pustulosis (AGEP), maculopapular rash, pretibial erythema with edema, and reversible cerebral vasoconstriction syndrome (RCVS). Although infrequent, these severe hypersensitivity and cardiovascular events pose significant clinical risks due to their association with substantial morbidity and, in some cases, mortality. The primary aim of this review is to consolidate available clinical evidence to evaluate the causality, characteristic clinical presentations, and broader safety implications of etoricoxib in relation to these adverse outcomes. While SJS/TEN are marked by widespread epidermal necrosis and detachment, FDE typically recurs at fixed sites with residual pigmentation. Hematological complications such as drug-induced (TP) and Drug-induced IHA have also been reported, presenting as sudden platelet decline or severe hemolysis, respectively. These adverse effects often appear within hours to weeks of initiating therapy. Cutaneous manifestations, including exanthematous pustulosis and maculopapular rashes, further complicate the drug's safety profile. Etoricoxib's pro-thrombotic potential, possibly linked to COX-2 selectivity, remains a cardiovascular concern. Causality assessments via the Naranjo Scale and WHO-UMC often support a probable link. These findings underscore the necessity for careful evaluation of patient history, immediate drug discontinuation upon clinical suspicion, and strengthened pharmacovigilance systems to better capture and characterize the full range of these rare yet serious reactions.

See also the graphical abstract(Fig. 1).

## Abbreviations

ADR: Adverse Drug Reactions

AF: Atrial Fibrillation

AGEP: Acute Generalized Exanthematous Pustulosis

BSA: Body Surface Area

COX: Cyclooxygenase

DAT: Direct Antiglobulin Test

FDE: Fixed Drug Eruptions

GBFDE: Generalized Bullous Fixed Drug Eruption

GI: Gastrointestinal

IAT: Indirect Antiglobulin Test

IHA: Immune Hemolytic Anemia

LTT: Lymphocyte Transformation Test

NSAIDs: Non-Steroidal Anti-Inflammatory Drugs

RCVS: Reversible Cerebral Vasoconstriction Syndrome

RR: Relative Risk

SCARs: Serious Cutaneous Adverse Reactions

SJS: Stevens - Johnson syndrome

TEN: Toxic Epidermal Necrolysis

TNF-α: Tumor Necrosis Factor-α

TP: Thrombocytopenia

## Introduction

Etoricoxib is a selective cyclooxygenase-2 (COX-2) inhibitor and a distinct member of the non-steroidal anti-inflammatory drugs (NSAIDs) class. By targeting COX-2, it suppresses prostaglandin synthesis and thereby interrupts the biochemical cascades responsible for inflammation and pain (Ali et al., 2023[3]). Since its patent in 1996 and clinical approval in 2002 (Zisis et al., 2024[160]), etoricoxib has been widely prescribed to manage a range of painful and inflammatory conditions, including osteoarthritis, rheumatoid arthritis, ankylosing spondylitis, chronic low back pain, acute gout flares, and postoperative dental pain (Grosser et al., 2011[60]).

The rationale for prescribing COX-2 selective inhibitors like etoricoxib has centered on their improved gastrointestinal (GI) safety compared to non-selective NSAIDs, which inhibit both COX-1 and COX-2 enzymes. By sparing COX-1, etoricoxib was expected to minimize gastric mucosal injury, a common drawback of non-selective NSAIDs (Wirth et al., 2024[148]). This perceived advantage positioned it as a safer alternative for patients with NSAIDs induced GI intolerance. However, growing clinical evidence and post-marketing surveillance have challenged this assumption, revealing the need to reexamine its safety profile, particularly concerning cardiovascular, renal, and dermatological risks (Harirforoosh et al., 2013[63]).

Although etoricoxib is known for its favorable GI tolerability, its broader safety profile remains under active scrutiny (Yeole et al., 2022[152]). Clinical case reports have highlighted a spectrum of ADRs, prompting reconsideration of the drug's risk-benefit balance, especially as its global use expands through both prescription and over-the-counter channels. Clinicians and patients may underestimate the drug's potential for severe or fatal outcomes due to its perceived safety. An increasing number of studies and case reports implicate etoricoxib in serious cutaneous adverse reactions such as TEN (Kameshwari and Devde 2015[75]), SJS (Ahamed et al., 2024[1]), and FDE (Apoorva et al., 2019[7]). Furthermore, cardiovascular events like new-onset atrial fibrillation (Kotgire et al., 2019[82]) and hypertensive crises (Challa et al., 2022[33]), as well as hematological complications including drug-induced IHA (Domingues et al., 2017[49]) and TP (Manappallil and Krishnan 2016[94]), have been reported. Other documented reactions include AGEP (Mäkelä and Lammintausta 2008[91]), pretibial erythema (Kumar 2015[85]), systemic maculopapular eruptions (Inamdar et al., 2017[71]), and RCVS (Dallocchio et al., 2014[42]). These ADRs may occur unpredictably and, in some cases, prove fatal, yet the literature lacks a consolidated synthesis of such data.

Despite the growing body of reports, the available literature remains fragmented and largely confined to isolated case descriptions. This review, therefore, aims to provide a systematically organized synthesis of dermatological, cardiovascular, hematological, and neurological ADR associated with etoricoxib. By compiling and analyzing published case reports, it seeks to support safer prescribing practices and encourage further investigation into the drug's evolving safety profile.

## Pharmacological Profile of Etoricoxib

### Mechanism of action

**Figure 2:**
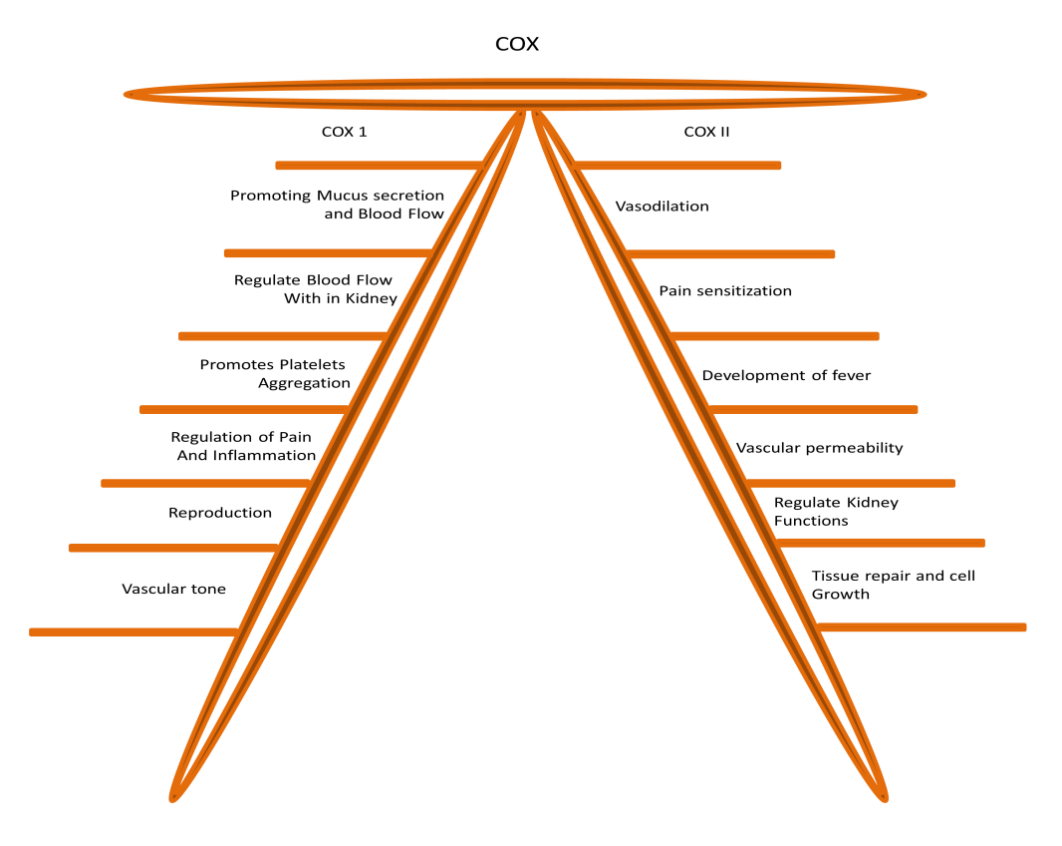
Physiological roles of COX-1 and COX-2

Etoricoxib differs from traditional drugs due to its high selectivity for the COX-2 isoform (Ali et al., 2023[3]). Quantitative studies have shown that etoricoxib exhibits approximately 30-fold greater selectivity for COX-2 over COX-1, a feature that shapes both its therapeutic profile and associated risks (Lenders et al., 2020[88]). Understanding its mechanism of action requires a clear distinction between the physiological roles of COX-1 and COX-2 (Figure 2(Fig. 2)). COX-1 is constitutively expressed and supports essential homeostatic functions, such as maintaining gastric mucosal integrity, ensuring renal perfusion, and facilitating platelet aggregation (Sakamoto 1998[125]; Morita 2002[103]; Bruno et al., 2023[24]). In contrast, COX-2 expression is inducible, primarily activated by inflammatory signals, and leads to the synthesis of pro-inflammatory prostaglandins (Yao and Narumiya 2019[150]; Wautier and Wautier 2023[146]).

**Figure 3:**
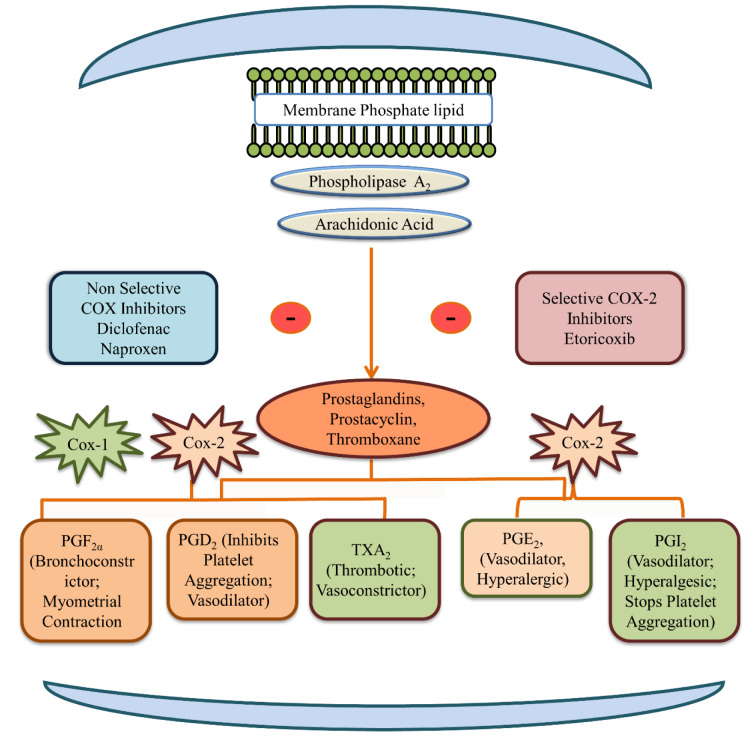
Pathway mediated by COX-1, COX-2 and inhibition by NSAIDs

By selectively inhibiting COX-2, etoricoxib prevents the conversion of arachidonic acid into inflammatory prostaglandins responsible for pain, swelling, and fever (Figure 3(Fig. 3)) (Kaur and Singh 2022[78]). This selective mechanism underlies its pharmacodynamic action as an anti-inflammatory and analgesic agent. Clinicians prescribe selective COX-2 inhibitors like etoricoxib based on the rationale that they preserve COX-1 mediated gastric protection, thereby minimizing GI toxicity. In contrast, non-selective NSAIDs inhibit both COX isoforms, reducing protective prostaglandins in the gastric mucosa and increasing the risk of ulceration, bleeding, and perforation (Tziona et al., 2017[140]). Etoricoxib's selectivity is thus hypothesized to offer GI safety without sacrificing efficacy. Clinical trials support this view, indicating a lower incidence of GI complications compared to traditional NSAIDs (Ramey et al., 2005[119]). Nonetheless, whether this benefit holds across all populations remains an open question, warranting further evaluation.

### Pharmacokinetics and pharmacodynamics

A comprehensive understanding of etoricoxib's pharmacokinetic and pharmacodynamic properties is essential for evaluating not only its therapeutic utility but also its potential to elicit adverse effects, particularly in at-risk populations. When administered orally, etoricoxib is rapidly absorbed, reaching peak plasma concentrations within approximately one hour (Renner et al., 2010[121]). Its oral bioavailability is nearly complete (~100 %), indicating efficient GI absorption with minimal first-pass hepatic metabolism (Shi and Klotz 2008[130]). After absorption, the drug demonstrates a large volume of distribution (~120 liters), suggesting extensive tissue penetration (Shah and Kotadiya 2022[128]). ~92 % of circulating etoricoxib binds to plasma proteins primarily albumin which may alter its distribution in conditions such as hypoalbuminemia (Takemoto et al., 2008[135]).

Etoricoxib undergoes hepatic metabolism, chiefly via cytochrome P450 enzymes, with CYP3A4 playing a dominant role (Kaur and Singh 2022[78]). The primary pathway includes 6′-methyl hydroxylation followed by further oxidation to a 6′-carboxylic acid metabolite, which constitutes the major urinary excretion product (Rodrigues et al., 2003[122]). While additional isoenzymes such as CYP2D6, CYP2C9, and CYP1A2 may contribute, but their roles appear to be secondary. These metabolites are pharmacologically inactive and exhibit negligible COX-1 inhibition, thereby limiting unintended effects on platelet function or gastric mucosa (Patrignani et al., 2003[114]; Takemoto et al., 2008[135]; Tsoupras et al., 2024[139]). Renal clearance (after i.v. administration) accounts for about 70 % of drug elimination, with the remainder excreted in feces (~20 %). Less than 1 % of etoricoxib is excreted unchanged. Its terminal half-life of around 22 hours permits once-daily dosing and facilitates steady-state plasma concentrations with chronic use (Rodrigues et al., 2003[122]; Takemoto et al., 2008[135]).Together, these pharmacokinetic attributes govern the systemic exposure to etoricoxib and carry clinical relevance.

### Therapeutic benefits and clinical use

Etoricoxib has established a distinct therapeutic niche in managing both acute and chronic pain, owing to its potent anti-inflammatory and analgesic actions paired with relatively favorable GI tolerability (Patrício et al., 2013[113]). Clinicians frequently prescribe it for degenerative joint disorders such as osteoarthritis and rheumatoid arthritis, as well as for axial spondyloarthropathies like ankylosing spondylitis (Kristensen et al., 2015[84]; Bittar and Deodhar 2025[20]). Its use also extends to acute inflammatory conditions, including gout flares, postoperative dental pain, primary dysmenorrhea, and chronic low back pain all of which involve significant inflammatory processes (Table 1(Tab. 1); References in Table 1: Balazcs et al., 2016[13]; Bickham et al., 2016[19]; Brooks and Kubler, 2006[22]; Brown et al., 2013[23]; Bruyère et al., 2016[25]; Daniels et al., 2011[44]; Malmstrom et al., 2003[93]; Moore et al., 2010[102]; Zhang et al., 2016[154]).

**Figure 4:**
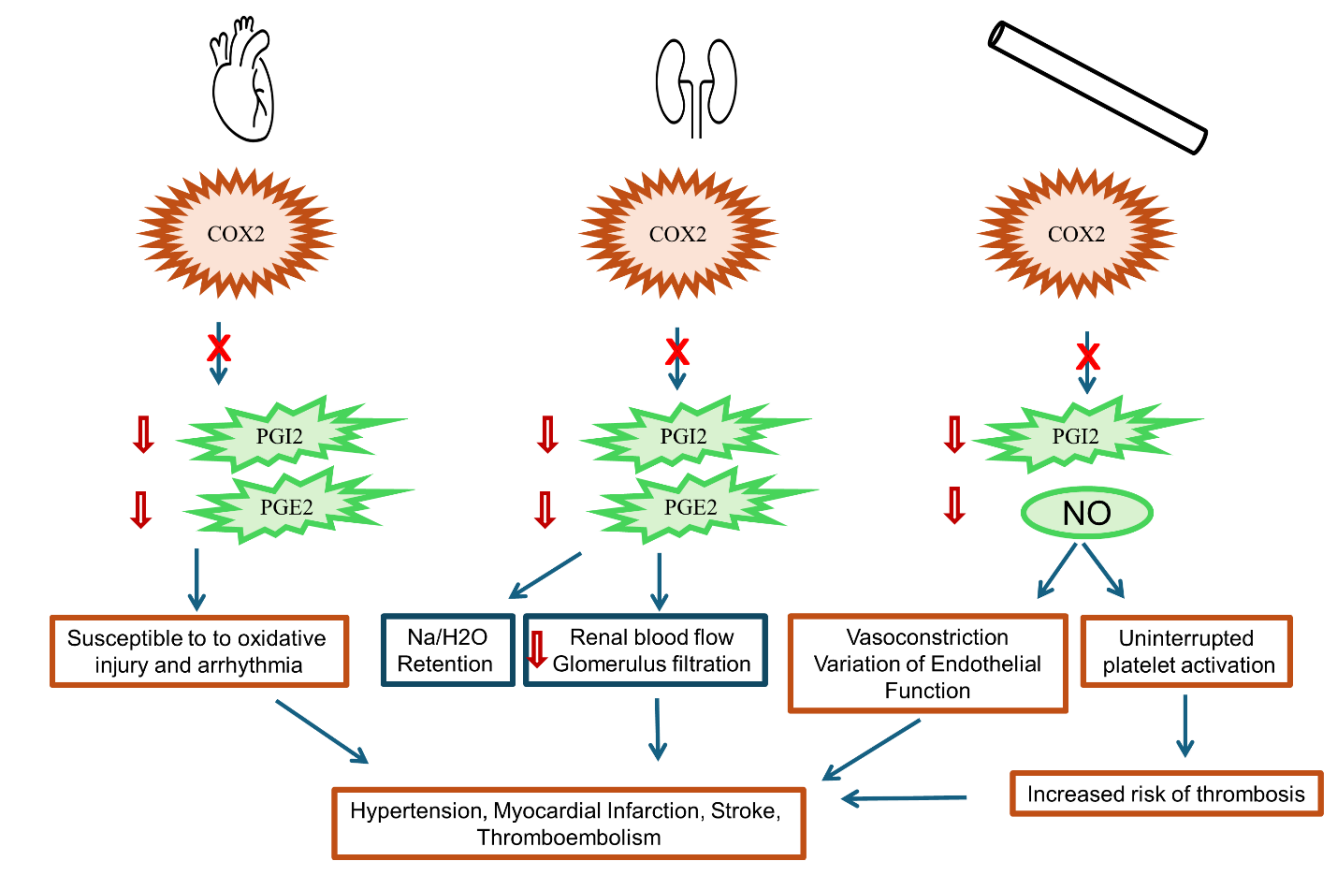
Cardio and nephrotoxicity pathway of etoricoxib

Although etoricoxib remains a valuable component in the pharmacological management of pain and inflammation, its benefit risk profile warrants contextualization within a broader clinical framework. Individuals with established cardiovascular disease, renal dysfunction, or fluid retention tendencies may require heightened surveillance or alternative pharmacologic approaches (Figure 4(Fig. 4)). Balancing efficacy against potential adverse outcomes necessitates individualized risk stratification. This consideration further justifies the need to investigate underreported but clinically significant adverse effects, which are discussed in the following sections of this review.

### SJS and TEN

SJS is a rare but potentially fatal mucocutaneous disorder marked by widespread epidermal necrosis and detachment, predominantly affecting the skin and mucous membranes. If not promptly recognized and managed, SJS can progress to life-threatening complications such as sepsis, multiorgan failure, and death (Shanbhag et al., 2020[129]). Medications, particularly antibiotics, antiepileptics, and NSAIDs, are commonly trigger, of SJS (Zhou et al., 2025[156]). In some cases, infections caused by the herpes simplex virus or the Epstein-Barr virus also initiate the reaction (Stanley et al., 2024[132]). TEN represents the severe end of the SJS-TEN spectrum, primarily characterized by extensive keratinocyte apoptosis and near-total epidermal detachment, typically following drug exposure (Bordeanu-Diaconescu et al., 2024[21]).

### Clinical presentation and diagnosis

SJS and TEN forms a spectrum of rare yet life-threatening mucocutaneous reactions, most often triggered by medications, and characterized by extensive keratinocyte apoptosis and necrosis (Zimmerman and Dang 2019[158]; Garg et al., 2023[55]). The extent of epidermal detachment distinguishes them: SJS affects less than 10 % of body surface area (BSA), TEN exceeds 30 %, while cases involving 10-30 % are categorized as SJS-TEN overlap (Cao et al., 2023[29]; Chuenwipasakul et al., 2023[39]). Early symptoms are often non-specific and flu-like fever, malaise, sore throat, and arthralgia typically emerging days before any cutaneous involvement (Marzano et al., 2016[98]; Dodiuk-Gad et al., 2017[48]).

Cutaneous findings usually begin as dusky, erythematous maculopapular rashes sometimes pruritic initially localized to the limbs, trunk, face, or neck, and later becoming generalized. These lesions can progress into vesicles and bullae, which coalesce and rupture, resulting in widespread epidermal sloughing that exposes the underlying tender dermis (Stredova et al., 2024[133]). The distribution is typically bilaterally symmetrical, affecting areas such as the trunk, extremities, face, scalp, genitalia, and occasionally the palms and soles. A hallmark clinical sign is the positive Nikolsky sign, where lateral pressure causes epidermal detachment (Wong et al., 2016[149]; Lerch et al., 2017[89]).

Mucosal involvement occurs in nearly 90 % of cases and serves as a crucial diagnostic criterion. The oral cavity, lips, conjunctiva, and genital mucosa are especially prone to involvement (Charlton et al., 2020[34]). Mucosal lesions may present as hemorrhagic crusts, erosions, and conjunctival injection; in severe cases, corneal ulceration or symblepharon may develop (Harr and French 2010[64]; Kohanim et al., 2016[80]). Systemic involvement is frequent, with complications including hematologic abnormalities (such as anemia and lymphopenia), renal dysfunction and hepatic injury (Creamer et al., 2016[40]; Kohanim et al., 2016[81]). Such multisystem involvement underscores both the clinical severity and the need for urgent intervention.

Diagnosis relies primarily on clinical evaluation, corroborated by a thorough drug history and the timeline of symptom onset. Histopathological examination can support diagnosis, often revealing full-thickness epidermal necrosis, subepidermal blister formation, and a sparse lymphocytic infiltrate (Frantz et al., 2021[53]; Garg et al., 2023[55]). To assess causality, clinicians frequently use structured tools like the Naranjo Scale, WHO-UMC criteria, and the ALDEN algorithm. Prognosis is guided by the SCORTEN score, which incorporates variables such as age, heart rate, serum bicarbonate, and urea levels (Teschke 2025[136]). Differential diagnosis should include other exfoliative conditions, notably Staphylococcal Scalded Skin Syndrome, which typically spares mucous membranes (Obeid et al., 2015[108]).

### Epidemiology and causality

The incidence of SJS and TEN in the general population remains low, estimated at approximately 1-2 cases per million annually for SJS and 2-5 cases per million for the combined SJS-TEN spectrum (Marks et al., 2023[95]; Castellana et al., 2025[31]). Etoricoxib, has only rarely been implicated in such reactions, isolated case reports have described etoricoxib-induced severe cutaneous adverse reactions, including erythema multiforme-like eruptions, AGEP, and, in rare instances, SJS or TEN. Nonetheless, post-marketing surveillance and available peer-reviewed literature suggest these occurrences are exceedingly infrequent (Martínez Antón et al., 2021[97]).

Determining causality in such adverse events is inherently complex. These reactions often arise unpredictably, particularly in settings of polypharmacy or underlying patient susceptibility. In many cases, the latency period between drug exposure and symptom onset generally within 1 to 3 weeks matches the expected timeline for T-cell mediated immunologic reactions (Kameshwari and Devde 2015[75]; Thakur and Lahiry 2019[137])

The potential involvement of genetic predispositions, particularly HLA polymorphisms such as HLA-B*15:02 or HLA-A*31:01, along with polymorphic variants in drug-metabolizing enzymes like CYP2C19, adds a layer of complexity to the risk assessment. While specific data on etoricoxib are limited, these molecular mechanisms have been implicated in serious cutaneous adverse reactions (SCARs) induced by related agents and may play a role here as well (Buchner et al., 2021[26]; Perelló et al., 2022[115]). Ultimately, although etoricoxib is generally considered safe for the majority of patients, the potential for rare but life-threatening cutaneous adverse effects demands continued pharmacovigilance. Early recognition of prodromal symptoms, timely discontinuation of the suspect drug, and patient education regarding early warning signs are critical components of clinical risk mitigation.

### Pathophysiological consideration

The immunopathogenesis of SJS and TEN centers on dysregulated T-cell-mediated responses directed against keratinocytes expressing drug-modified antigens (Cheng 2021[36]; Gibson et al., 2024[58]). These are classified as type IV (delayed-type) hypersensitivity reactions more specifically, type IVc driven primarily by cytotoxic CD8⁺ T lymphocytes. Upon exposure to a triggering agent, drug metabolites or hapten-protein complexes can alter keratinocyte surface markers, thereby initiating an aberrant immune response. Cytotoxic CD8⁺ T cells subsequently infiltrate the epidermis and release a cascade of pro-apoptotic mediators, including perforin, granzyme B, and granulysin. Among these, granulysin has emerged as a key effector molecule in the widespread keratinocyte apoptosis characteristic of TEN (Harris et al., 2016[65]; Bellón 2019[17]; Hung et al., 2024[69]).

Concurrently, Fas-FasL (CD95-CD95L) interactions activate the caspase cascade, further driving programmed cell death, while cytokines such as Tumor Necrosis Factor-α (TNF-α) and IFN-γ exacerbate local tissue damage and systemic inflammation. Keratinocytes themselves may contribute to the cytotoxic loop by releasing TNF-α and expressing Fas ligand, amplifying both autocrine and paracrine pathways (Su and Chung 2014[134]). Recruitment of additional immune cells including CD4⁺ T cells and dermal macrophages augments the dermo epidermal interface injury. Emerging evidence also implicates IL-15 and CXCL10 in sustaining inflammatory infiltration and systemic immune activation (Bellón et al., 2022[18]).

Although direct mechanistic studies involving etoricoxib remain limited, these immunopathological processes are presumed to be relevant. Etoricoxib's pharmacokinetic profile, particularly its hepatic metabolism and interaction with transporter systems, may indirectly modulate immune activation. Whether this pharmacokinetic interaction alone precipitates SCARs remains speculative, but it aligns with the multifactorial etiology observed in SJS and TEN.

### Etoricoxib induced TEN and SJS-TEN overlap syndromes

The literature reveals a growing number of SCARs attributed to etoricoxib, with several well documented cases of TEN and SJS-TEN overlap. These cases not only reflect the unpredictable immunopathological responses to COX-2 inhibitors but also underscore the need for early recognition and individualized supportive interventions (Table 2(Tab. 2); References in Table 2: Kameshwari and Devde 2015[75]; Kreft et al., 2010[83]; Pandey et al., 2022[110]; Rachana et al., 2015[118]; Roy et al., 2018[123]; Thakur and Lahiry 2019[137]).

### SJS and related milder presentations

While the aforementioned cases typically fulfilled criteria for TEN or SJS-TEN overlap based on BSA involvement, additional reports reflect SJS phenotypes, generally involving <10 % of BSA but with significant mucocutaneous morbidity. In these cases, causality was consistently rated (Table 3(Tab. 3); References in Table 3: Ahamed et al., 2024[1]; Kumar and Tharuni, 2021[86]; Zisis et al., 2024[160]). 

## FDE

### Clinical features

A distinct, immunologically mediated cutaneous drug reaction, characterized by the recurrence of lesions at the same anatomical sites upon re-exposure to a specific medication. While FDE often presents as a solitary lesion, multiple fixed lesions are not uncommon, especially with repeated exposures or prolonged administration of the offending agent (Anderson et al., 2021[5]; McClatchy et al., 2022[101]).

Clinically, lesions in FDE are usually well circumscribed, round or oval erythematous plaques. However, variations in morphology particularly in mucosal variants can occur (Pretzlaff et al., 2015[116]). These lesions often display a dusky or violaceous hue and may appear as flat macules, edematous plaques, or more complex formations with vesicles or bullae (Banerjee 2017[15]). In well-established sites, bullae may rupture, resulting in erosions or ulcerations, especially in the oral and genital mucosa. Patients frequently describe symptoms such as pruritus, burning, or mild discomfort associated with the lesions (Cheraghlou and Levy 2020[37]).

### Diagnosis 

A key diagnostic hallmark of FDE is its temporal pattern. The initial eruption may manifest several days to two weeks after first exposure to the causative drug. However, upon subsequent exposures, lesions typically reappear more rapidly often within 24 hours or even just a few hours highlighting the role of immunological memory and the persistence of drug-specific T cells localized to previously affected skin (Özkaya 2013[109]; Anderson et al., 2021[5]; Schunkert et al., 2021[127]).

Post-inflammatory hyperpigmentation is another defining feature of FDE. This residual pigmentation at the site of the previous lesion may persist for months or longer. Notably, re-exposure usually triggers recurrence at these pigmented sites, often with increased severity. While the hallmark of FDE is recurrence at identical sites, new lesions may also develop over time, particularly in cases of ongoing or intermittent exposure (Maghfour et al., 2022[90]). Although FDE can affect any part of the skin or mucosal surfaces, it shows a predilection for areas such as the lips, oral mucosa, genitalia (especially the glans penis, scrotum, and perianal region), and common cutaneous locations including the face, neck, limbs, trunk, palms, and soles. Mucosal involvement is common but not obligatory (Pretzlaff et al., 2015[116]; Mortazavi et al., 2022[104]).

A more severe variant, known as Generalized Bullous Fixed Drug Eruption (GBFDE), presents with multiple well-demarcated bullous lesions affecting more than three anatomical sites. While its clinical presentation can resemble SJS or TEN, GBFDE tends to follow a more benign course, with minimal or absent systemic involvement and a comparatively favorable prognosis (Cho et al., 2014[38]). Classic FDE typically lacks systemic features such as fever, and even in GBFDE, these manifestations are usually mild or absent (Patel et al., 2020[112]).

From a diagnostic standpoint, clinical history and lesion morphology are critical. The reappearance of lesions at previously affected sites after re-administration of the drug particularly when accompanied by post-inflammatory pigmentation is highly suggestive (Lee 2000[87]; Pretzlaff et al., 2015[116]). Diagnostic support can be obtained through adjunctive methods. Among these, patch testing preferably performed on hyper pigmented. However, sensitivity is variable and false negatives are not uncommon (de Groot 2022[45]). The oral challenge test which entails re-administering the suspected drug in a controlled setting, is regarded as the most definitive diagnostic tool. A positive test is indicated by lesion recurrence within 24 hours (Imbesi et al., 2010[70]). Due to the potential risk of severe reactions, especially in GBFDE or cases with extensive involvement, the oral challenge test must be used cautiously and is typically contraindicated in high-risk patients (Esperouz et al., 2025[52]).

Histopathological evaluation through skin biopsy can aid diagnosis in atypical or uncertain cases. Characteristic histologic features include hydropic degeneration of basal keratinocytes, necrotic keratinocytes, pigment incontinence, and perivascular lymphocytic infiltrates, often with melanophages reflecting past inflammation. In bullous forms, subepidermal blistering may be evident (Weyers and Metze 2011[147]; Kauppinen and Kariniemi 2020[77]; Patel et al., 2020[112]). Although not routinely required in straightforward presentations, biopsy remains valuable in differentiating FDE from other dermatoses such as erythema multiforme or SJS (Patel et al., 2020[112]). Other diagnostic techniques, such as the lymphocyte transformation test (LTT), have been investigated for identifying culprit drugs in FDE. Nevertheless, due to its limited sensitivity, technical demands, and inconsistent clinical utility, the LTT has not gained widespread adoption (McClatchy et al., 2022[101]).

Based on these clinical and pathological insights, the following section presents concise summaries of reported cases of etoricoxib-induced FDE (Table 4(Tab. 4); References in Table 4: Andrade and Gonãalo 2011[6]; Apoorva et al., 2019[7]; Augustine et al., 2006[12]; Calistru et al., 2011[28]; Carneiro-Leão and Rodrigues Cernadas 2019[30]; De Sousa et al., 2016[46]; Duarte et al., 2010[50]; Kaomongkolgit et al., 2019[76]; Makris et al., 2024[92]; Movsisyan et al., 2019[105]; Ponce et al., 2012; Siriwattanasatorn et al., 2025[131]; Vera et al., 2021[142]).

Researchers documented seven patients who developed FDE linked to etoricoxib, each exhibiting distinct yet overlapping clinical signs suggestive of etoricoxib-specific hypersensitivity. The cohort comprised four female and three male patients, aged between 35 and 77 years. All cases showed recurrent erythematous or vesiculobullous lesions, which typically reappeared at the same anatomical locations following drug re-exposure. Lesions were distributed across various regions, including the eyelids, flanks, axillae, groin, cheeks, neckline, and forearms; less typical areas such as the scalp and scapular region were also affected in some cases. Diagnostic confirmation was achieved mainly through lesional patch testing with etoricoxib 10 % in petrolatum, yielding positive responses in six of the seven patients. A persistent feature across cases was post-inflammatory hyperpigmentation, coupled with the recurrence of lesions at fixed anatomical sites. Three patients demonstrated multilocular involvement. Cross-reactivity testing, performed in patients, showed no hypersensitivity to conventional NSAIDs such as ibuprofen, ketoprofen, naproxen, or paracetamol. Notably, celecoxib was well tolerated in two patients, with patch tests returning negative outcomes (Martínez Antón et al., 2021[97]).

## Cardiotoxicity and Thrombotic Risk

### COX-2 selectivity and cardiovascular implications

The rationale behind developing COX-2 selective inhibitors, or coxibs, was to suppress inflammation driven prostaglandin production without compromising COX-1 mediated physiological functions, especially those safeguarding the GI tract thereby reducing the GI toxicity common to non-selective NSAIDs (Knights et al., 2010[79]).

Yet, this pharmacological advancement was soon clouded by concerns over cardiovascular safety. In the early 2000s, the withdrawal of rofecoxib and Valdecoxib following increased reports of myocardial infarction and other cardiovascular events sparked regulatory alarm (Atukorala and Hunter 2013[11]). The U.S. Food and Drug Administration subsequently declined to approve etoricoxib, citing an unfavorable benefit-risk profile, particularly with respect to myocardial infarction risk. These regulatory developments intensified scrutiny of both selective and non-selective NSAIDs, revealing that elevated risks of acute myocardial infarction may be a class effect, though the magnitude varies according to agent, dose, and duration of exposure (Zingler et al., 2016[159]).

Mechanistically, the prevailing "thrombotic imbalance" hypothesis implicates COX-2 selectivity in disrupting the equilibrium between prostacyclin (PGI₂) and thromboxane A₂ (TXA₂). PGI₂, produced by endothelial COX-2, exerts vasodilatory and antithrombotic effects. In contrast, TXA₂ synthesized via COX-1 in platelets promotes vasoconstriction and platelet aggregation. Selective inhibition of COX-2 reduces PGI₂ levels without affecting TXA₂ synthesis, thus potentially tipping the balance toward a pro-thrombotic state (Grosser 2006[59]). Etoricoxib may therefore pose a comparatively greater cardiovascular risk.

Nonetheless, this mechanistic model does not fully account for the clinical heterogeneity observed across different NSAIDs. Other pathophysiological factors may contribute independently or synergistically to cardiovascular risk. These include disruptions in endothelial nitric oxide synthase activity, modulation of renal sodium retention, and elevations in systemic blood pressure (Ghosh et al., 2015[57]). 

### Clinical evidence and meta-analyses

Randomized trials, cohort studies, and meta-analyses present a complex and sometimes inconsistent understanding of etoricoxib's cardiovascular risk. Although aggregated data generally suggest that coxibs pose a higher relative cardiovascular risk than non-selective NSAIDs, the magnitude and relevance of this risk remain context-specific. A comprehensive meta-analysis showed a modest but significant increase Relative Risk (RR) in cardiovascular events associated with NSAIDs overall (RR ~1.24), with coxibs (RR ~1.22) displaying slightly higher risks than non-selective NSAIDs (RR ~1.18). The relative risk for etoricoxib (RR ~1.27) positioned it near diclofenac and rofecoxib, which are already known for heightened cardiovascular risk profiles (Martín Arias et al., 2019[96]). Similarly, a Chinese cohort study revealed a more than twofold increased hazard ratio (HR 2.01) for composite cardiovascular disease in etoricoxib users, raising concerns about population-specific vulnerability or prescribing practices (Wan et al., 2023[144]). However, the findings are not uniformly consistent. In contrast to observational studies, a network meta-analysis of randomized trials did not find sufficient evidence to implicate etoricoxib in elevated myocardial infarction (MI) risk compared to placebo. Nonetheless, the same analysis reported a high probability that etoricoxib, like many NSAIDs, raises the overall risk of major cardiovascular events by over 30 % (Trelle et al., 2011[138]). This discrepancy underscores a critical limitation: randomized trials frequently exclude high-risk individuals and may lack the power to detect rare but serious outcomes.

The association between etoricoxib and stroke remains equivocal, with conflicting data emerging from diverse populations. While ibuprofen and diclofenac have consistently shown a stronger linkage to stroke risk, the evidence implicating etoricoxib is both limited and contradictory. Some East Asian cohort studies suggest an elevated risk of ischemic stroke among etoricoxib users (Wan et al., 2023[144]). These inconsistencies likely reflect differences in baseline health status, comorbid conditions, or prescribing habits. Atrial fibrillation (AF), once under recognized, has also been linked to etoricoxib use (Table 5(Tab. 5); References in Table 5: Challa et al., 2022[33]; Kotgire et al., 2019[82]) (Zheng et al., 2025[155]). Regulatory authorities and clinical consensus both emphasize caution in prescribing etoricoxib to patients with known cardiovascular disease. In such populations, alternatives should be considered, or the lowest effective dose should be used for the briefest possible duration. Continued pharmacovigilance and large-scale comparative studies are essential to refine our understanding of etoricoxib's cardiovascular safety in real-world settings.

## Thrombocytopenia

### Clinical presentation

Drug-induced TP is an infrequent yet clinically consequential adverse effect that can emerge suddenly or gradually following the administration of various pharmacologic agents, including NSAIDs (Kam and Alexander 2014[74]). Although only a limited number of cases implicate etoricoxib, growing evidence suggests that it can provoke severe TP, often accompanied by mucocutaneous bleeding (Table 6(Tab. 6); References in Table 6: Manappallil and Krishnan 2016[94]; Recinella et al., 2019[120]) (Recinella et al., 2019[120]; Nagi et al., 2021[106]). Patients typically present with features of a bleeding diathesis, ranging from minor signs such as petechiae and purpura to more serious manifestations like mucosal hemorrhage, hematuria, or GI bleeding. Laboratory assessments uniformly demonstrate profound reductions in platelet counts, occasionally reaching critically low levels and raising concern for major hemorrhagic events (Priziola et al., 2010[117]). 

### Pathophysiological considerations

Drug-induced TP arises through a multifaceted pathogenesis that includes both immune-mediated and non-immune mechanisms. TP can occur due to heightened peripheral platelet destruction, reduced production in the bone marrow, or sequestration within the spleen. Among drug-induced causes, immune-mediated platelet destruction is recognized as the principal mechanism. A key immunological pathway involves the generation of drug-dependent antibodies that bind to platelets only in the presence of the causative drug, forming immune complexes (Aster et al., 2009[10]). Certain drugs act as haptens by covalently attaching to platelet surface proteins and triggering a humoral immune response, while others stimulate the formation of autoantibodies that cross-react with native platelet antigens, thereby mimicking the immunopathogenesis of idiopathic thrombocytopenic purpura (Immunohematology and 2014). Alternatively, some chemotherapeutic agents and antibiotics induce TP through direct myelotoxic effects that suppress megakaryocyte activity and reduce platelet production (Vo and Thompson 2019[143])

### Diagnosis

Diagnosing drug-induced TP demands heightened clinical vigilance, particularly when TP arises without a clear cause after recent drug initiation. In the absence of pathognomonic features, diagnosis remains largely clinical anchored on temporal association with drug exposure, exclusion of secondary causes, and rapid platelet recovery upon withdrawal of the suspected agent. A detailed clinical history is indispensable, with close attention to drug exposures within the preceding one to two weeks, consistent with the latency of immune-mediated reactions. Initial laboratory evaluation should include a complete blood count, peripheral smear, and coagulation profile, while secondary causes such as viral infections, autoimmune conditions, and hematologic malignancies must be excluded (Danese et al., 2020[43]). If anemia is present, a direct Coombs' test may help rule out Evans syndrome (Dhingra et al., 2008[47]). Ultimately, the resolution of TP within 24-48 hours after drug discontinuation provides the most definitive evidence of drug-induced TP, while continued exposure may rapidly escalate to life-threatening hemorrhage.

## Immune Hemolytic Anemia

### Clinical presentation

Etoricoxib-induced IHA represents a rare yet clinically consequential complication linked to various pharmacological agents (Table 7(Tab. 7); References in Table 7: Burgos Pratx et al., 2019[27]; Domingues et al., 2017[49]; Mayer et al., 2013[100]). Its onset is often acute, though in certain cases, symptoms may evolve gradually over several days (Hill et al., 2017[67]). Patients frequently report nonspecific systemic symptoms such as increasing fatigue, profound adynamia, and intermittent nausea. On physical examination, pallor is a common finding; in more severe anemia, a soft systolic murmur may be detected, reflecting hemodynamic compensation (Salama 2009[126]). 

### Pathophysiological considerations

The immunopathogenesis of drug-induced IHA hinges on the formation of drug-dependent antibodies that typically become reactive only in the presence of the causative agent or its metabolites. These antibodies may form circulating immune complexes with the drug, which then bind to red blood cell (RBC) membranes, triggering complement activation and subsequent hemolysis. This mechanism contrasts with classical warm auto IHA, where autoantibodies target intrinsic RBC antigens independent of drug exposure. The conditional nature of antibody binding in drug-induced IHA introduces significant diagnostic complexity, particularly when standard serological assays are performed without the implicated drug present (Garratty 2010[56]).

Several mechanistic pathways have been proposed to explain the immunological basis of drug-induced IHA, each reflecting a different interaction between drug molecules, RBCs, and the host immune system. First, in hapten-mediated reactions, the drug covalently binds to RBC surface proteins, forming hapten-carrier complexes that the immune system recognizes as foreign. Second, in autoantibody-type responses, the drug induces antibodies that cross-react with native RBC antigens, essentially mimicking primary Auto IHA. Third, the ternary complex mechanism involves a simultaneous interaction among the drug, specific antibodies, and membrane components often requiring all three to initiate a hemolytic cascade. Lastly, non-immunologic protein adsorption describes a scenario in which the drug nonspecifically modifies the RBC membrane, promoting abnormal protein binding that can provoke immune clearance (Habibi 2019[61]; Baldo and Pham 2021[14]).

### Diagnosis

Immune-hematological testing plays a central role in diagnosis. The direct antiglobulin test (DAT) is nearly positive in drug-induced IHA (Johnson et al., 2007[73]), with findings frequently demonstrating C3d reactivity alongside variable presence of immunoglobulins such as IgG, IgM, and IgA (Mayer et al., 2015[99]). The indirect antiglobulin test (IAT), while less consistently positive, may show pan-reactivity or nonspecific binding that complicates interpretation (Arndt and Garratty 2005[8]). Taken together, these immunohematologic findings are essential for distinguishing drug-induced IHA from other causes of hemolytic anemia.

## Other Reported Adverse Conditions

AGEP, etoricoxib induced maculopapular rash with systemic involvement, pretibial erythema with edema linked to etoricoxib, RCVS.

The occurrence and severity of etoricoxib-related adverse effects are not uniformly distributed across the patient population; rather, several predisposing factors can increase an individual's susceptibility to these undesirable outcomes. These factors often relate to pre-existing comorbidities, demographic characteristics, concomitant medications, and the duration or dosage of etoricoxib administration (Table 8(Tab. 8); References in Table 8: Dallocchio et al., 2014[42]; Inamdar et al., 2017[71]; Kumar 2015[85]; Mäkelä and Lammintausta 2008[91]).

A documented case series by Edel et al., describes eleven patients (seven males and four females; mean age 45 years, range 25-81) who developed oral mucosal lesions following administration of etoricoxib, predominantly at a dosage of 90 mg/day for pain control. Clinically, the majority of patients (8/11) presented with acute, painful erosions localized to the hard palate, although the tongue, buccal mucosa, and lips were also involved in several cases. Notably, one patient exhibited cutaneous target lesions, raising concern for a more extensive mucocutaneous hypersensitivity reaction. In ten of the eleven individuals, cessation of etoricoxib followed by topical or systemic corticosteroid therapy resulted in complete resolution within 10 to 21 days.

Rechallenge with etoricoxib was performed in six patients, all of whom experienced a reproducible recurrence of symptoms, thereby reinforcing the causal association. The temporal proximity of symptom onset to drug administration and the characteristic erosive mucosal pattern strongly point toward a Type B hypersensitivity reaction, likely immune-mediated in nature. Importantly, none of the patients reported prior intolerance to other NSAIDs, suggesting that this reaction may not be a manifestation of NSAID-class cross-reactivity. A distinctive diagnostic feature observed across cases was the consistent palatal predilection of lesions, though initial misdiagnosis was common largely attributed to a lack of prescriber familiarity with this specific adverse reaction profile (Edel et al., 2019[51]).

## Discussion

Etoricoxib, a COX-2 inhibitor, influences prostaglandin production and aims to spare COX-1 (which protects renal function), potentially leading to adverse renal and cardiovascular effects (Figure 3(Fig. 3)) (Ahmadi et al., 2022[2]). NSAIDs cause increases in sodium and water retention, particularly pronounced with COX-2 inhibitors, contributing to elevated blood pressure (Challa et al., 2022[32]). Etoricoxib-induced cardiotoxicity is marked by alterations in arachidonic acid metabolism and beta-adrenergic signaling pathways. It upregulates cyp4a12, beta-1 adrenergic receptor (adrb1), and cyp2j5, while downregulates cyp2c29, ace2 gene expression, leading to cardiac diseases and drug-induced cardiotoxicity. NSAID-induced inhibition of prostaglandin formation can lead to vasoconstriction and reduced blood supply, contributing to observed effects like hyaline degeneration of cardiac muscle fibers (Banu et al., 2020[16]; Pascale et al., 2021[111]; Askar et al., 2022[9]; Jamous et al., 2024[72]). Inhibition of COX-2 in cardiomyocytes may directly contribute to cardiovascular hazards and affect cardiac rhythm (Wang et al., 2009[145]).

SJS and TEN are severe, potentially fatal skin disorders characterized by generalized keratinocyte necrosis due to inappropriate immune activation (Hama et al., 2024[62]). They are primarily classified as Type IV (subtype C) hypersensitivity reactions, indicating a cell-mediated immune response. The core pathophysiology involves CD8+ T-lymphocytes and activated macrophages in the epidermis, alongside CD4+ T-cells in the dermis, suggesting a cytotoxic cellular immune reaction targeting keratinocytes (Hausmann et al., 2010[66]). Etoricoxib or its metabolites may act as haptens, binding to keratinocytes and rendering them antigenic, which then triggers the immune response. This leads to the activation of apoptotic pathways, notably the CD95 ligand (Fas ligand) and TNF-α systems, resulting in widespread keratinocyte death (Obeid et al., 2015[108]). In cases of SJS-TEN overlap involving etoricoxib and methotrexate, a pharmacokinetic interaction is debated, where etoricoxib might inhibit the elimination of methotrexate (via HOAT-3 transporter inhibition in renal tubules), leading to its accumulation to toxic levels and potentially contributing to the severe reaction (Rachana et al., 2015[118]). 

FDE is characterized as a delayed-type localized hypersensitivity reaction. Similar to SJS/TEN, Drug-specific CD8+ T-cells are activated and differentiate into resident memory T-cells that persist within the epidermis of the affected skin sites. Re-exposure to etoricoxib may rapidly reactivate these resident CD8+ memory T-cells, leading to a copious release of pro-inflammatory cytokines, such as IFN-γ and TNF-α (Makris et al., 2024[92]). This cytokine release, along with cytotoxic molecules (granzyme B, perforin) from CD8+ T-cells, causes localized inflammation and tissue damage, primarily targeting keratinocytes and melanocytes, resulting in epidermal necrosis and the characteristic lesions. CD4+ T-cells are also involved, with regulatory T-cells migrating into the lesions and potentially helping to limit harmful immune reactions and contribute to spontaneous resolution (Yawalkar et al., 2000[151]; Hiroyasu et al., 2021[68]).

Etoricoxib-induced DIIHA may involve drug-dependent antibodies that react with red blood cells (RBCs) in the presence of the drug or its metabolites. These antibodies can activate the complement system, leading to hemolysis (Mayer et al., 2013[100]). DITP typically involves increased platelet destruction, likely immune-mediated. The mechanism can involve drug-dependent antibodies forming immune complexes that then react with platelets, causing their destruction (Vayne et al., 2020[141]). Etoricoxib-induced pleural effusion is not entirely clear, but proposed mechanisms include fluid retention, dose-dependent toxic effects, chemical inflammation, or oxidative stress of mesothelial cells (Sah et al., 2023[124]). Etoricoxib has been linked to extensive oral ulceration. These reactions are often described as erosive, aphthous-like, or erythema multiforme-like and are considered a "Type B reaction," which implies an idiosyncratic, often immune-mediated, response (Kaomongkolgit et al., 2019[76]).

Genetic predisposition plays a significant role in an individual's susceptibility to certain adverse effects. Specific Human Leukocyte Antigen (HLA) alleles are known to influence susceptibility to severe cutaneous adverse reactions (Zhu et al., 2024[157]). For example, HLA-B*15:02 and HLA-B*15:11 alleles are associated with an elevated risk of developing SJS when using carbamazepine, particularly among Asian populations (Amstutz et al., 2014[4]). HLA-B*58:01 has been linked to allopurinol-induced SJS in both Asian and non-Asian individuals (Ng et al., 2016[107]). Other implicated HLA alleles include HLA-A*31-01, HLA-A*24:02, and HLA-B*13:01 (Fricke-Galindo et al., 2017[54]).

An underlying defect in the detoxification system of keratinocytes can also play a major role in drug-induced SJS/TEN. Polymorphisms in the CYP2C19 gene, which encodes a cytochrome P450 isoform, can also heighten the susceptibility to SJS when exposed to certain medicines like phenobarbital, phenytoin, or carbamazepine (Yip and Pirmohamed 2022[153]). A specific polymorphism, the brain-derived neurotrophic factor (BDNF) Val66Met polymorphism, has been linked to vasoconstriction in patients with RCVS (Chen et al., 2011[35]). This indicates that genetic variations influencing drug metabolism can predispose individuals to these severe reactions.

The effectiveness of treatment options for adverse effects associated with etoricoxib largely depends on the specific adverse effect, but a common and crucial first step across many conditions is the immediate discontinuation of etoricoxib. In SJS and TEN, immediate withdrawal of the drug is crucial. Supportive intensive therapy is essential, including aggressive fluid resuscitation and electrolyte maintenance. This often leads to prompt or rapid improvement. Antibiotics (prophylactic or therapeutic) are typically initiated. A combination of linezolid and clindamycin was found effective in one case (Thakur and Lahiry 2019[137]). Cyclosporine was added in one case due to its ability to promote shorter reepithelialization time and lower mortality, leading to a prompt recovery (Thakur and Lahiry 2019[137]), but one case did not improve symptoms, leading to collapse after 5 days (Roy et al., 2018[123]). High-dose systemic glucocorticoids may have a favorable effect only in the initial days of exanthema development, with later use potentially increasing sepsis risk (Kreft et al., 2010[83]; Rachana et al., 2015[118]). Dexamethasone, prednisolone, and methylprednisolone (parenteral or oral) were used in several cases, leading to rapid improvement and healing of lesions (Kreft et al., 2010[83]; Kameshwari and Devde 2015[75]; Rachana et al., 2015[118]; Pandey et al., 2022[110]; Makris et al., 2024[92]; Zisis et al., 2024[160]). Infliximab, a TNF-α antibody, showed evident and rapid efficacy in one case of TEN; a "single-shot" administration resulted in the progression of epidermolysis stopping within a few hours, with re-epithelization achieved within 5 weeks (Kreft et al., 2010[83]).

For FDE, topical corticosteroids (e.g., fluocinolone, acetonide) are commonly used to reduce symptoms like itching and inflammation, and can accelerate healing (Kaomongkolgit et al., 2019[76]; Kumar and Tharuni, 2021[86]). Oral corticosteroids (e.g., prednisolone, methylprednisolone, and betamethasone) are prescribed for more severe or extensive lesions, also leading to significant improvement or complete healing (De Sousa et al., 2016[46]; Apoorva et al., 2019[7]; Kaomongkolgit et al., 2019[76]; Makris et al., 2024[92]; Siriwattanasatorn et al., 2025[131]). Oral antihistamines (hydroxyzine) can provide symptomatic relief from itching (Apoorva et al., 2019[7]). Mouthwashes (e.g., chlorhexidine, gentian violet) can be used for oral erosions (Kaomongkolgit et al., 2019[76]). Ceftriaxone, fluticasone, and gentian violet are also used (Apoorva et al., 2019[7]).

In IHA, steroid therapy (Prednisolone) (Mayer et al., 2013[100]), erythrocyte concentrates (Domingues et al., 2017[49]) were utilized. In one severe case, intravenous methylprednisolone and immunoglobulin (i.v. gamma globulin) were used, leading to complete resolution of the anemia (Burgos Pratx et al., 2019[27]). In DITP also the complete withdrawal of the offending drug is the fundamental step. Recovery of platelet count often occurs within 1 to 2 days of discontinuation. Platelet transfusions are indicated in cases of severe thrombocytopenia (e.g., wet purpura) due to high bleeding risk (Manappallil and Krishnan 2016[94]). Corticosteroids and intravenous immunoglobulin have been used (Recinella et al., 2019[120]). 

Elevations in blood pressure are usually short-lived and reverse once the drug is discontinued, achieving adequate blood pressure control. One patient's blood pressure normalized without needing other antihypertensive therapy after etoricoxib withdrawal. If etoricoxib use is essential or blood pressure remains high, antihypertensive therapy may be added and monitored (Challa et al., 2022[33]). In a reported case of AF, the patient reverted to a sinus rhythm after treatment (metoprolol, amiodarone, aspirin, enoxaparin, and bisoprolol), which included drug discontinuation (Kotgire et al., 2019[82]). In oral Ulceration, withdrawal of the drug is the immediate action. Palliative therapy with systemic corticosteroids (e.g., prednisone, dexamethasone rinse, clobetasol cream) and topical treatments (e.g., fluocinolone acetonide in orabase, chlorhexidine mouthwash) is used. The oral ulceration often significantly improves and completely heals after a few weeks (e.g., 4 weeks) with drug withdrawal and palliative therapy (Edel et al., 2019[51]; Kaomongkolgit et al., 2019[76]).

The effectiveness of preventing adverse effects associated with etoricoxib relies on a multi-faceted approach, central to which are careful patient selection, appropriate monitoring, the strategic use of alternative medications, and comprehensive patient education. A crucial first step in prevention is a thorough review of the patient's medical history before prescribing etoricoxib. Etoricoxib, despite its efficacy in pain management and enhanced gastrointestinal tolerability, has a safety profile that is still being explored and is a matter of debate. Etoricoxib should be used with caution in patients with pre-existing renal or cardiac conditions, including uncontrolled hypertension, ischemic heart disease, or heart failure (Jamous et al., 2024[72]). A history of hypersensitivity or immunosuppression may increase the chances of developing serious mucocutaneous reactions. Clinicians should inquire about past drug reactions and must consider the risk of drug-drug interactions (DDIs), especially when prescribing low-dose methotrexate concurrently with etoricoxib. Etoricoxib can inhibit the elimination of methotrexate, potentially leading to toxic accumulation (Rachana et al., 2015[118]).

Regular and diligent monitoring of patients receiving etoricoxib is vital for early detection and prevention of severe complications. Regular monitoring of blood pressure is essential in patients prescribed etoricoxib, especially those with pre-existing hypertension. If etoricoxib use is essential in patients with blood pressure >140/90 mmHg, antihypertensive therapy should be added and blood pressure monitored for two weeks after starting and regularly thereafter. Elevated blood pressure levels are usually short-lived and reverse once the drug is de-challenged (Challa et al., 2022[33]).

Selecting appropriate alternatives is crucial. A simpler analgesic like paracetamol may be used as a first-line agent for common conditions like back pain, rather than newer, unfamiliar agents like etoricoxib (Siriwattanasatorn et al., 2025[131]). Patient education is critical to prevent recurrence and ensure patient safety. Patients must be informed about the potential for adverse reactions to etoricoxib, even if rare. Patients should be advised to report any unwanted side effects as soon as possible. It is crucial to instruct patients to completely avoid the causative drug and, potentially, structurally related drugs, especially for reactions like FDE, SJS, and TEN, as re-exposure can lead to more severe lesions and prolonged recovery, or even fatal outcomes. Patients should be provided with an allergy identification card and be advised on the risks of self-medication. More studies, including clinical trials, are needed to fully elucidate the risks and mechanisms associated with etoricoxib-induced toxicities, such as nephro- and cardiotoxicity observed in animal models.

## Conclusion

This analysis has systematically reviewed the dermatological adverse effects associated with etoricoxib, specifically focusing on SJS, TEN, and FDE, etoricoxib induced atrial fibrillation and hypertension**, **etoricoxib induced TP, etoricoxib induced IHA, AGEP, etoricoxib induced maculopapular rash, pretibial erythema with edema linked to etoricoxib, and RCVS. The overarching aim was to synthesize existing clinical evidence regarding the manifestation, diagnosis methods, and treatment, of these rare yet severe reactions, evaluating whether the available data consistently supported a causative link.

The key findings unequivocally demonstrate that etoricoxib, while generally well-tolerated, can indeed precipitate in the studied reactions. In the context of SJS/TEN, the research indicates that these reactions are rare but life-threatening mucocutaneous conditions, with reported cases consistently exhibiting the hallmark widespread keratinocyte apoptosis and epidermal detachment. The clinical presentation often began with non-specific prodromal symptoms, rapidly progressing to extensive cutaneous and mucosal involvement, necessitating urgent clinical management. Similarly, FDE emerged as a distinct, recurrent, site-specific erythematous or bullous lesion upon re-exposure to etoricoxib, frequently leaving residual hyperpigmentation. Both SJS/TEN and FDE exhibited characteristic temporal associations, with symptom onset ranging from hours to a few weeks following etoricoxib administration. The available case studies suggest a discernible, albeit modest, risk of etoricoxib induced atrial fibrillation and hypertension. Lastly, our analysis confirms that etoricoxib can, in rare instances, precipitate significant hematological complications, specifically etoricoxib induced TP and etoricoxib induced IHA, highlighting a broader systemic reactivity. Other less common but distinct adverse events, such as AGEP, maculopapular rash, pretibial erythema with edema, and RCVS, further contribute to the drug's nuanced safety profile. Causality assessment tools such as the Naranjo Scale, WHO-UMC criteria, and ALDEN algorithm, employed in numerous documented cases, consistently assigned a "probable" relationship, reinforcing the direct support for these conclusions based on the presented evidence. Furthermore, positive oral challenges and lesional patch tests for FDE cases provided compelling evidence of drug-specific hypersensitivity.

The broader implications of these findings for clinical practice and pharmacovigilance are considerable. While the incidence of etoricoxib induced adverse reactions remains low, their severity necessitates heightened clinical vigilance. Clinicians ought to maintain a high index of suspicion when patients present with new dermatological symptoms, particularly in the context of etoricoxib use, especially if there is a history of polypharmacy, autoimmune comorbidities, or prior hypersensitivity reactions. The recurrence of FDE upon re-exposure underscores the critical importance of careful patient history-taking and explicit advice on drug avoidance. Furthermore, the documented cases highlight the ongoing need for robust pharmacovigilance reporting to capture and analyze such rare adverse events comprehensively. Future research might reasonably focus on identifying specific genetic predispositions or predictive biomarkers that could stratify patient risk, potentially allowing for more personalized prescribing practices and mitigating the occurrence of these profound dermatological sequelae associated with etoricoxib. This synthesis contributes to the ongoing scholarly conversation concerning the risk-benefit profile of selective COX-2 inhibitors, emphasizing that even rarely observed adverse events can have significant patient impact.

## Declaration

### Funding information

Not applicable.

### Authors contribution

MAA: Writing original draft, Methodology, Investigation, Conceptualization. AK: Supervision, Conceptualization, Resources.

### Ethics approval 

Not applicable.

### Declaration of competing interest

The authors state no conflicts of interest related to this investigation.

### Consent to participate 

Not applicable. 

### Acknowledgment

The authors extend their sincere gratitude to the Graphic Era Hill University. 

### Artificial Intelligence (AI) - Assisted Technology

The authors also acknowledge the use of language support tools, including ChatGPT and Grammarly, for refining the clarity and readability of the manuscript.

## Figures and Tables

**Table 1:**
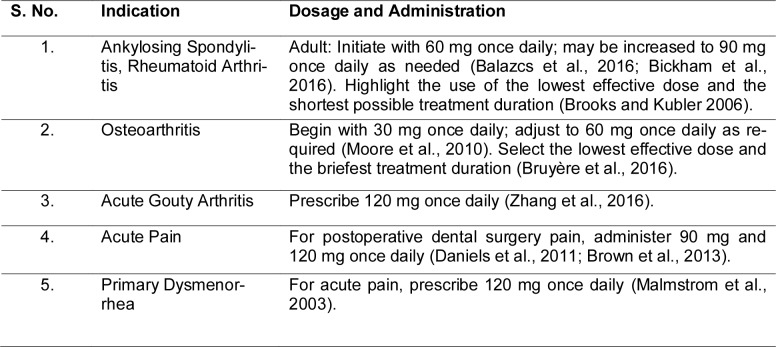
Dosage and indication of etoricoxib

**Table 2:**
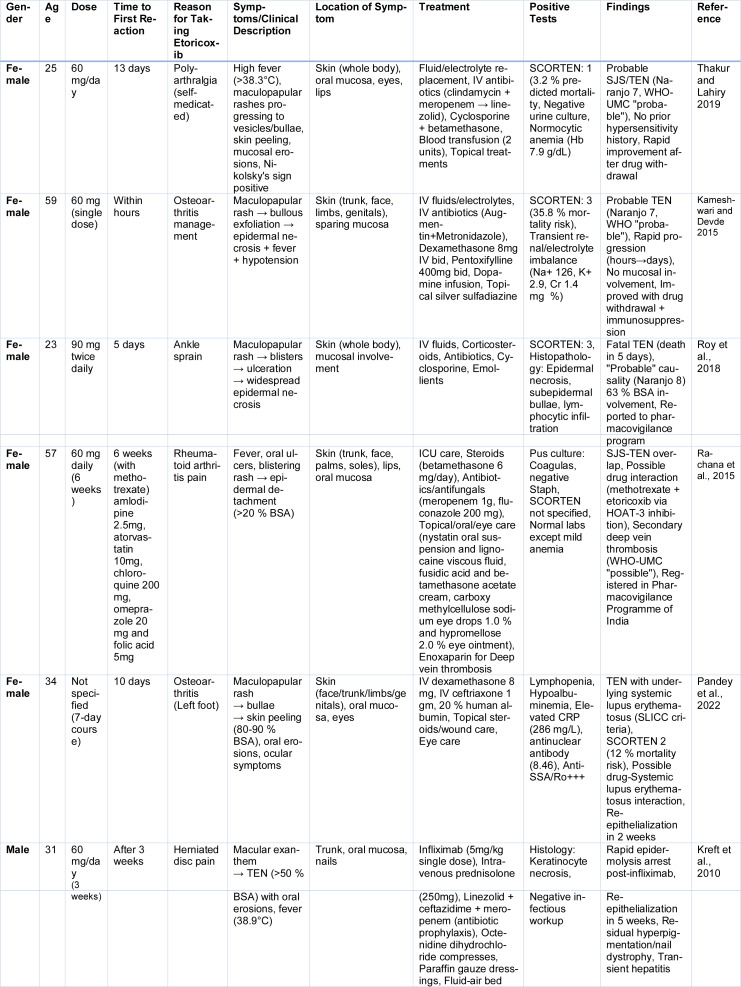
Cases reported on Etoricoxib induced TEN and SJS-TEN Overlap

**Table 3:**
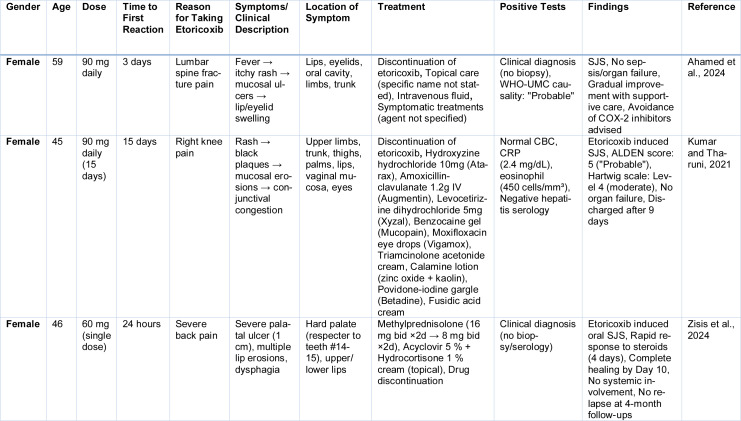
Cases reported of Etoricoxib induced SJS

**Table 4:**
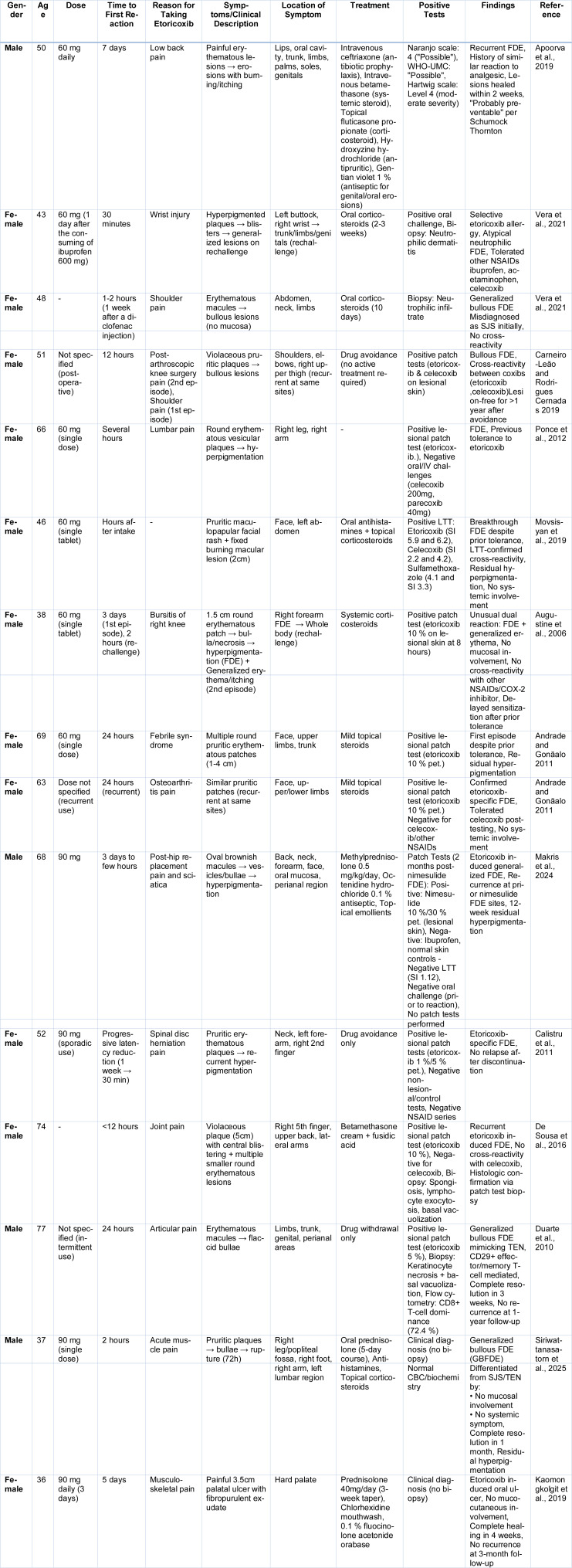
Cases reported on Etoricoxib induced FDE

**Table 5:**
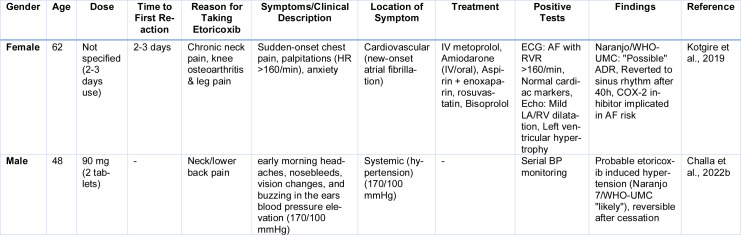
Cases reported on Etoricoxib induced AF and hypertension

**Table 6:**
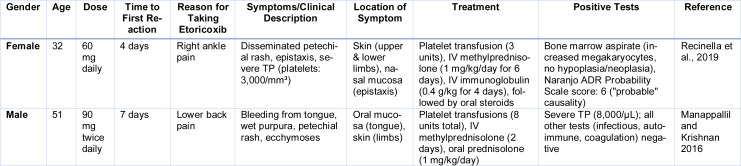
Cases reported on Etoricoxib induced Thrombocytopenia

**Table 7:**
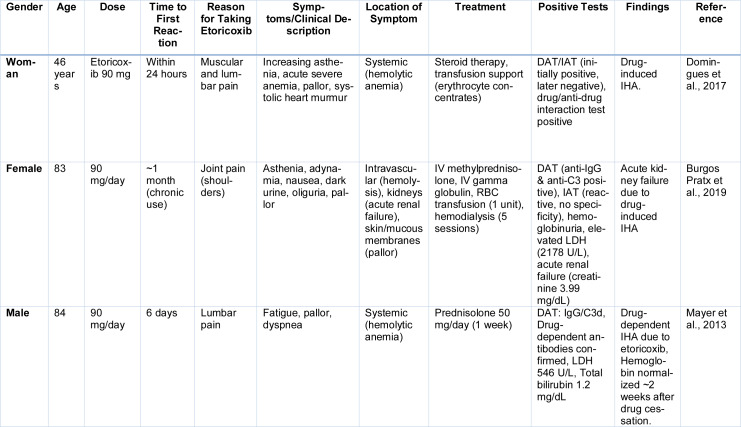
Cases reported on Etoricoxib induced Immune Hemolytic Anemia

**Table 8:**
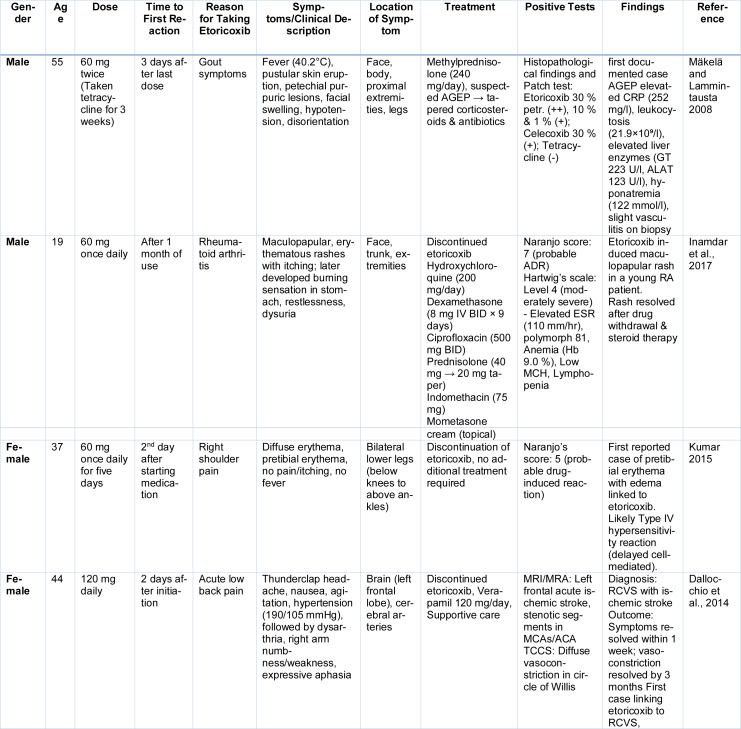
Cases reported on Etoricoxib induced AGEP, maculopapular rash, pretibial erythema and RCVS with ischemic stroke

**Figure 1:**
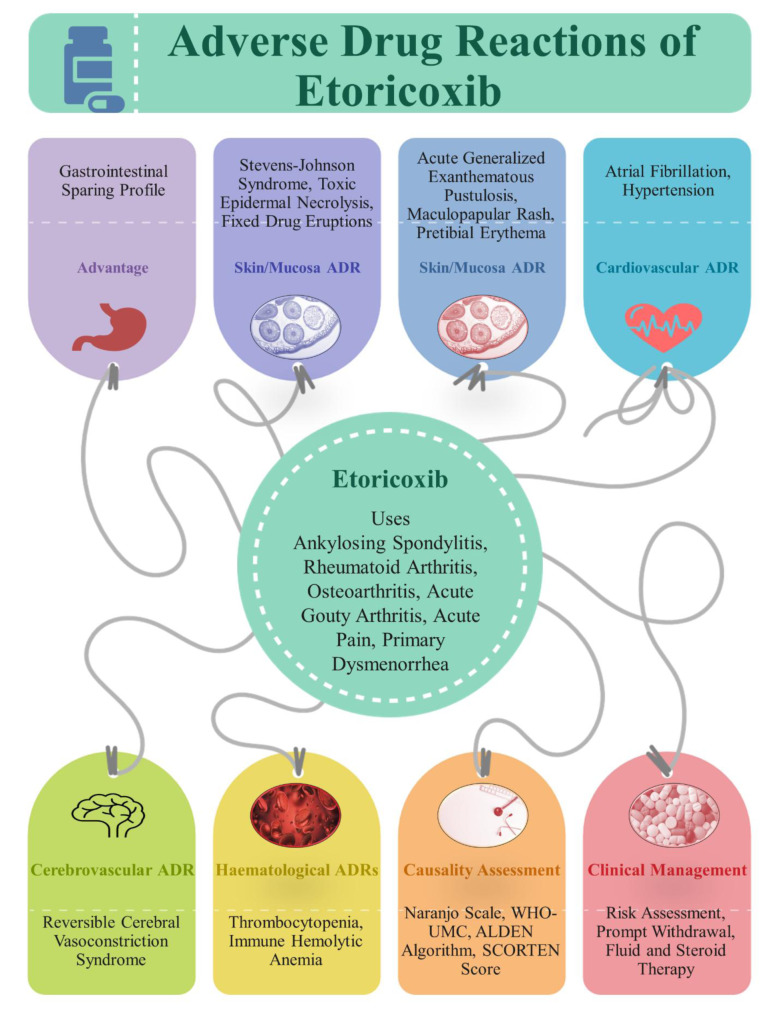
Graphical abstract
